# Exosome–Autophagy Crosstalk in Enveloped Virus Infection

**DOI:** 10.3390/ijms241310618

**Published:** 2023-06-25

**Authors:** Yuqi Wang, Linzhu Ren, Haocheng Bai, Qing Jin, Liying Zhang

**Affiliations:** Key Lab for Zoonoses Research, College of Animal Sciences, Ministry of Education, Jilin University, Changchun 130062, China; wangyuqi21@mails.jlu.edu.cn (Y.W.); renlz@jlu.edu.cn (L.R.); baihc20@mails.jlu.edu.cn (H.B.); jinqing22@mails.jlu.edu.cn (Q.J.)

**Keywords:** extracellular vesicles, autophagy, relationship, viruses

## Abstract

Exosomes, which are extracellular vesicles (EVs) predominantly present in bodily fluids, participate in various physiological processes. Autophagy, an intracellular degradation mechanism, eliminates proteins and damaged organelles by forming double-membrane autophagosomes. These autophagosomes subsequently merge with lysosomes for target degradation. The interaction between autophagy and endosomal/exosomal pathways can occur at different stages, exerting significant influences on normal physiology and human diseases. The interplay between exosomes and the autophagy pathway is intricate. Exosomes exhibit a cytoprotective role by inducing intracellular autophagy, while autophagy modulates the biogenesis and degradation of exosomes. Research indicates that exosomes and autophagy contribute to the infection process of numerous enveloped viruses. Enveloped viruses, comprising viral nucleic acid, proteins, or virions, can be encapsulated within exosomes and transferred between cells via exosomal transport. Consequently, exosomes play a crucial role in the infection of certain viral diseases. This review presents recent findings on the interplay between exosomes and autophagy, as well as their implications in the infection of enveloped viruses, thereby offering valuable insights into the pathogenesis and vaccine research of enveloped virus infection.

## 1. Introduction

Exosomes originate from multivesicular bodies (MVBs) and are present in various body fluids, including blood, saliva, urine, and breast milk. In 1983, Pan et al. discovered the secretion of vesicles from immature red blood cells in sheep [1], which were later referred to as “exosomes” by Johnstone et al. in 1987 [2]. These exosomes are formed within MVBs and transported to the plasma membrane where they merge with the membrane and are subsequently released into the extracellular space [3]. Numerous studies have demonstrated that different cell types can secrete exosomes, which contribute to immune responses, antigen presentation, cell migration and differentiation, tumor invasion, and autophagy [4,5,6]. Recent investigations focusing on viral emergence and infection have revealed that exosomes can encapsulate the nucleic acids, proteins, and even virions of enveloped viruses, enabling their transmission from one cell to another through exosomal transport. Consequently, exosomes play a crucial role in the development of certain viral diseases.

Autophagy occurs within unique smooth two-membrane structures called autophagosomes which engulf cytoplasmic materials and subsequently fuse with lysosomes for degradation within the autophagosome–lysosome system [7]. Autophagy is a self-degradative process that maintains cellular homeostasis and promotes cell survival. It is a fundamental mechanism present in most eukaryotic cells under normal conditions. By breaking down cytoplasmic materials such as proteins and aged organelles, autophagy facilitates the renewal of cellular components [8]. During stressful conditions like starvation, hypoxia, or ER stress, autophagy is induced to provide energy and nutrients. This process acts as a self-protective mechanism for energy production and biosynthesis by degrading organelles, soluble proteins, into amino acids in the cytoplasm. Furthermore, autophagy eliminates misfolded or denatured proteins as well as aging or damaged organelles to maintain cellular homeostasis [9]. Aberrant autophagy can lead to the accumulation of toxic substances within cells, ultimately resulting in cell death. Studies have revealed a reciprocal regulation between the exosome and autophagy pathways, where their coordinated control plays a vital role in maintaining cellular homeostasis. Exosomes can modulate autophagy through various mechanisms, while autophagy, in turn, impacts the formation and release of exosomes. Additionally, targeting key signaling pathways and associated molecules involved in autophagy can reduce the release of detrimental exosomes, thereby influencing the occurrence and progression of various diseases.

## 2. Similarities and Differences between Exosomes and Autophagy Biogenesis

Exosomes are naturally occurring vesicles that bud inward from the membrane of late endosomes or MVBs, forming intraluminal vesicles (ILVs) [10,11]. As ILVs accumulate, they mature into MVBs, which subsequently fuse with the plasma membrane, leading to the release of luminal vesicles into the extracellular space. Consequently, the exosome membrane resembles the plasma membrane of the parent cell and is enriched with proteins and lipids associated with endosomal compartments [10,11]. The precise mechanism underlying exosome packaging remains incompletely understood; however, their secretion involves the formation of an Endosomal Sorting Complex Required for Transport (ESCRT) [12]. Late endosomes sequentially recruit ESCRT-0, -I, -II, -III, and VPS4 complexes. Through the assistance of the ESCRT complex, endosomal membranes invaginate, resulting in the generation of ILVs and subsequent formation of MVBs. The release of exosomes into the extracellular environment necessitates the transport and docking of MVBs, followed by their fusion with the cytoplasmic membrane [13]. Nevertheless, it has been demonstrated that only specific MVBs are capable of fusing with the plasma membrane. B lymphocytes can categorize MVBs into two groups based on their cholesterol content, with only MVBs exhibiting high cholesterol levels being capable of fusing with the plasma membrane and releasing exosomes [14]. Further investigations have highlighted the involvement of CD63 in exosome biogenesis, as the knockdown of CD63 using CRISPR/Cas9 technology has been shown to reduce exosome secretion [15]. Conversely, COS cells transfected with a small integral membrane protein of lysosome/late endosome (SIMPLE, also known as lipopolysaccharide-induced TNF-α factor, LITAF) exhibited increased exosome secretion, while mutations in SIMPLE disrupted normal formation of MVBs [16]. Additionally, both proteins and lipids play critical roles in vesicle transport, working in close cooperation for processes such as membrane deformation, fission, and fusion [17]. Studies have also revealed the enrichment of certain miRNAs within exosomes, suggesting that miRNAs can be loaded into these vesicles [18,19,20,21]. The uptake of cytosolic components during exosome biogenesis is a tightly regulated process. Research has indicated that exosomes lack argonaute (Ago) proteins and other nuclear or cytosolic genes involved in miRNA regulation, including Drosha, DGCR8, Dicer, TRBP, and GW182 [22]. Consequently, exosomes do not carry the necessary molecules for miRNA biogenesis outside the cell. It is important to note that the cargo loading of exosomes and the precise pathways governing their fusion and release with the plasma membrane remain areas requiring further investigation.

Furthermore, yeast studies have identified at least 38 genes encoding autophagy-related proteins (ATGs), many of which exhibit functional conservation in eukaryotes [23]. Autophagy initiation is triggered by the ULK-1-ATG13-FIP200 induction complex. The core autophagy proteins involved in the assembly and elongation of autophagosomes can be classified into two binding systems. The first system includes ATG3, ATG4, ATG7, as well as the ubiquitin-like modifiers MAP-LC3A, B, C (microtubule-associated protein light chain), GABA-RAP (γ-aminobutyric-acid-type-A-receptor-associated protein), and GATE-16 (Golgi-associated ATPase enhancer of 16 kDa). The second system comprises ATG5, ATG12, and ATG16, which colocalize with early autophagy structures [24]. In yeast, ATG8 acts as a regulator of phagosome expansion and is associated with autophagosome size. Reduction in ATG8 levels leads to an increase in smaller autophagosomes and a decrease in autophagy activity [25]. Additionally, ATG8 participates in cargo replenishment of autophagosomes through interaction with cargo-binding proteins like ATG19 [26]. ATG8 covalently binds to phosphatidylethanolamine (PE) on the autophagosome membrane, anchoring itself and activating autophagy. This process occurs in tandem with ATG4, ATG7, ATG3, and the ATG12-ATG5-ATG16L1 complexes [27].

In mammals, autophagy is predominantly regulated by the mammalian target of the rapamycin (mTOR) pathway. Abundant nutrients and growth factors stimulate the phosphorylation and inactivation of the autophagy initiation kinase ULK1 by mTOR complex 1 (mTORC1), thereby suppressing autophagy. Conversely, autophagy is induced by mTORC1 inactivation resulting from starvation conditions [28]. Moreover, the ATG1 and ULK1 complex initiates the nucleation of nascent phagophores [29]. Subsequently, two ubiquitin-like systems composed of ATG5-ATG12 and ATG7-ATG3 complexes covalently attach microtubule-associated protein LC3B to phosphatidylethanolamine on the autophagosome membrane [30,31]. Following autophagosome formation, they are transported to lysosomes. The outer autophagosome membrane and the lysosomal membrane then undergo fusion, forming an autolysosome. Within the autolysosome, the autophagosome membrane and sequestered materials undergo degradation, and the resulting degradation products are transported back to the cytoplasm for recycling [32]. Table 1 summarizes the key distinctions between exosomes and autophagy.

## 3. Crosstalk between Exosomes and Autophagy

**Figure 1:**
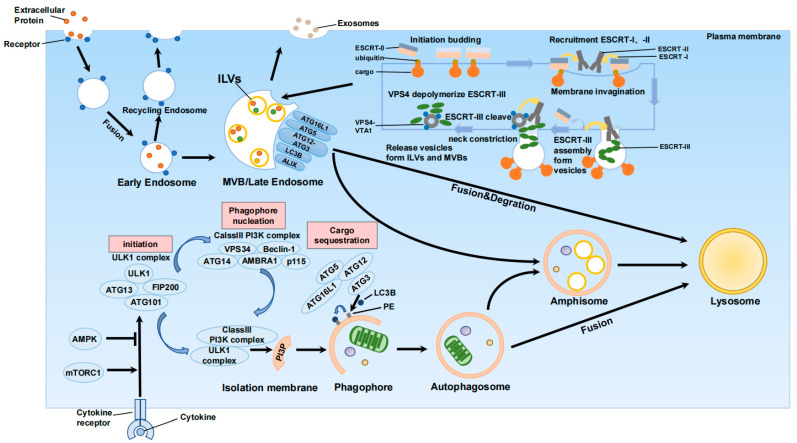
The relationship between exosomes and autophagy biogenesis occurs at different stages.

The intricate interplay between exosome and autophagy biogenesis takes place at various stages (Figure 1). 

Exosomes bud inward through the limiting membrane of late endosomes or multivesicular bodies (MVBs) to form intracavitary vesicles (ILVs), which fuse with the plasma membrane. The core autophagy proteins are involved in autophagosome assembly and extension mechanism which are mainly divided into two binding systems. The first binding system includes Atg3, Atg4, Atg7, ubiquitin-like modifiers MAP-LC3A, B, C (microtubule associated protein light chain), GABARAP and GATE-16. The second binding system includes Atg5, Atg12 and Atg16L1. These subpopulations of the autophagy mechanism have been proved to contribute to exosome biogenesis. Atg5 and Atg16L1 play a key non-autophagy function in exosome biogenesis. The form of MVBs is changed through the interaction between Atg12-Atg3 and Alix. Alix also reduces the basic autophagy flux and indicates the mutual regulation between autophagy and exosome biogenesis.

Initially, specific subsets of the autophagy machinery have been implicated in exosome biogenesis [33,34]. Moreover, dysfunctional MVBs can undergo degradation through autophagy. Inhibition of lysosomal activity or autophagy can restore exosome secretion [35]. Additionally, knockdown of the autophagy-related proteins ATG5 or ATG16L1 significantly diminishes exosome release and hampers the enrichment of lipidated LC3B within exosomes. This suggests that ATG5 and ATG16L1 play crucial non-autophagic roles in exosome biogenesis [34]. The ATG5-ATG16 complex facilitates the lipidation of non-classical LC3B and dissociates the V-type proton pump, preventing acidification of MVBs and lysosomal degradation. Consequently, it promotes fusion between MVBs and the plasma membrane, facilitating exosome release. Furthermore, treatment with lysosomal or V-ATPase inhibitors restores exosome release in ATG5 knockout cells, further confirming the impact of luminal pH on lysosomal degradation and MVB-plasma membrane fusion, as well as the role of autophagy-related proteins in exosome biogenesis [34]. 

Simultaneously, LC3B serves as a primary marker for autophagy flux. During the initiation stage, the ULK-mediated activation of the LC3B-binding complex triggers its modification and processing via ubiquitin-like systems, including ATG7 and ATG3, leading to autophagosome formation. At the maturation stage, LC3B facilitates autophagosome closure, fusion, and trafficking [36]. LC3B-associated phagocytosis (LAP), a process in which LC3B integrates into the phagosome membrane without autophagosome formation, has also been observed [37,38]. However, studies have shown that knockdown of ATG7 does not impact exosome release, indicating that autophagosome formation or LC3B lipidation is not essential for exosome biogenesis. These findings highlight the direct regulation of MVB fate and exosome biogenesis by autophagy-related proteins [34]. Furthermore, the protein ALIX is involved in membrane scission and interacts with ESCRT members to facilitate exosome release. Interaction between ATG12-ATG3 and ALIX alters MVB morphology, disrupts late endosome transport, and reduces exosome biogenesis. Concurrently, inhibition of ALIX diminishes primary autophagy flux, suggesting a reciprocal regulation between autophagy and exosome biogenesis [33].

Furthermore, the process of amphisome formation involves the fusion of MVBs, which consist of multiple vesicles, with autophagosomes. Subsequently, these amphisomes merge with lysosomes to facilitate the degradation of their contents. It is worth noting that there exists an antagonistic relationship between autophagy and exosome release, specifically in terms of amphisome degradation. For instance, when erythrocytic leukemia cell line K562 was subjected to starvation or treated with rapamycin, autophagy was induced, leading to an increase in the fusion of autophagosomes with MVBs, and, subsequently, a reduction in exosome release. This phenomenon resulted in re-autophagic degradation of MVBs [39]. Concurrently, studies have demonstrated that autophagic degradation of MVBs can be anticipated under various environmental conditions [35,40].

## 4. Role of Exosomes and Autophagy Crosstalk in Enveloped Virus Infection

**Figure 2:**
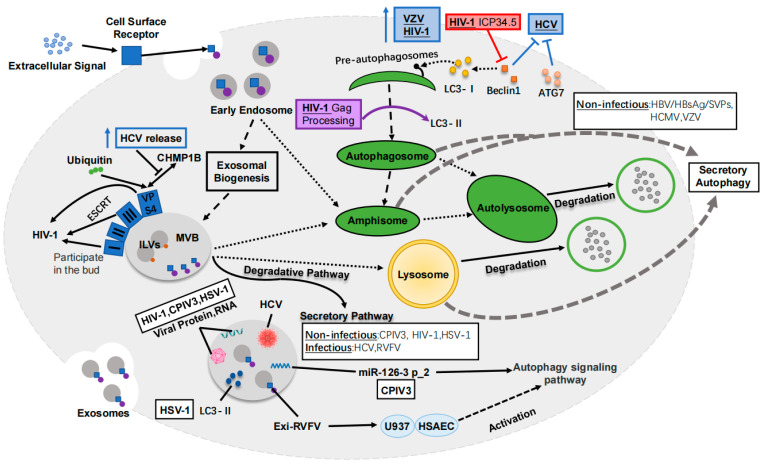
Exosomes and autophagy crosstalk in enveloped virus infection.

Recent research has provided evidence that exosomes can encapsulate and transfer nucleic acids, proteins, and enveloped virus particles between cells. In this intricate process, ESCRTs play crucial roles in cytokinesis, autophagy, retroviral budding, exosome formation and release, and other biological activities [41,42,43,44,45,46]. For instance, HIV utilizes the host cell’s ESCRT system to facilitate its budding, while enveloped viruses exploit the exosomal ESCRT system to enhance virus proliferation, budding, and dissemination [47,48,49,50,51]. Additionally, some viruses exploit autophagy to promote their replication and release [52]. Through autophagy, viral particles are able to access the extracellular space either through exosomes or viral envelopes. For instance, flaviviruses frequently employ autophagic membranes for replication and exocytosis via MVBs [53]. Investigations have revealed that viruses may utilize the interplay between autophagy and exosomes to facilitate their replication and release. Both the exosome release pathway and the secretory autophagy pathway can secrete viral products [54]. The interplay between exosomes and autophagy plays a pivotal role in the infection process of various enveloped viruses, thereby offering promising avenues for mechanistic research and treatment strategies targeting enveloped virus infections (Figure 2).

HIV-1 hijacks ESCRT-I, -III, and Vps4 to participate in its bud, without the need for ESCRT-0 and -II. HIV-1 induces autophagosome formation, while HIV-1 Nef blocks autophagosome lysosomal fusion in macrophages by interacting with Beclin 1. HIV-1 Gag colocates with LC3, and it may promote the degradation of viral protein Gag through ubiquitination. HCV promotes the polyubiquitination of VPS4A, thereby enhancing the interaction between VPS4A and CHMP1B. Beclin1 or ATG7 affects the level of extracellular vesicle related HCV, and the increase in autophagosome lysosomal fusion reduces the release of hepatitis C virus particles. CPIV3 infection can lead to EVs inhibiting autophagy and then inhibiting viral infection. The autophagy pathway plays a role in vesicle-mediated HSV-1 transmission.

### 4.1. RNA Viruses

#### 4.1.1. Human Immunodeficiency Virus (HIV)

According to the Joint United Nations Programme on HIV/AIDS (UNAIDS), the global death toll from acquired immune deficiency syndrome (AIDS)-related illnesses reached an estimated 42.2 million by 2019 [55]. Although combination antiviral therapy (cART) effectively suppresses plasma HIV-1 levels in most infected individuals, the virus persists in latent reservoirs established during the early phase of infection [56]. HIV can exploit the exosome pathway to evade the host immune system and enhance its infectivity. Retroviral budding, particularly in the case of HIV, resembles the biogenesis of exosomes [57]. HIV-1 utilizes the ESCRT system for budding, and VPS4 recruitment is crucial for this process in all ESCRT-dependent viruses [58]. Additionally, HIV-1 exploits ESCRT-I, -III, and Vps4 components to facilitate its own replication [59]. Conversely, autophagy acts as a defense mechanism against HIV. HIV-1 Gag colocalizes with LC3 [53], followed by ubiquitination which triggers the degradation of the viral protein Gag—an essential structural protein involved in virion assembly and release [60]. 

Moreover, mTOR can enhance the selective elimination of HIV-1-infected myeloid and T-cell populations by inhibiting viral transcription/translation and inducing autophagy, illustrating the interplay between EVs and autophagy [61]. Additionally, cannabidiol (CBD) significantly reduces the release of EVs by infected cells, likely through reduced viral transcription and activation of autophagy [62]. Furthermore, fully functional autophagy has been observed in HIV-infected human lymphoid aggregation cultures (HLACs). Modulating autophagy with agents that enhance autophagy (e.g., rapamycin) or inhibit it (e.g., 3-methyladenine, chloroquine, and bafilomycin) can reduce HIV-DNA levels and viral replication in these cells [63]. Notably, several key autophagic factors, such as BECN1, ATG5, ATG7, and LC3-II, were found to be elevated in the postmortem brains of HIV-1 encephalitis patients compared to HIV-1 patients without encephalitis [64].

Thus, it can be inferred that exosomes and autophagy interact through the interplay of related proteins and pathways in the context of HIV infection, impacting the course of the disease and providing novel therapeutic possibilities for HIV-1 treatment.

#### 4.1.2. Hepatitis C Virus (HCV)

Hepatitis C virus (HCV) is a blood-borne pathogen that can lead to liver fibrosis, hepatocellular carcinoma, and liver-related mortality. Despite the absence of a protective vaccine, HCV elimination relies on the treatment of existing infections and prevention of new ones [65]. During HCV infection, the ROS/JNK/Itch signaling pathway induced by the virus promotes the ubiquitination of VPS4A, facilitating its interaction with CHMP1B and increasing VPS4A ATPase activity. Consequently, this process enhances the release of HCV particles [66]. HCV infection also triggers an upregulation of autophagy, leading to the release of virus-containing exosomes [67,68]. Knockdown of Beclin1 or ATG7 has been shown to reduce the levels of HCV-associated extracellular exosomes, indicating the involvement of core autophagy mechanisms in packaging HCV particles into exosomes [69]. Furthermore, an increase in autophagosome–lysosome fusion results in reduced HCV particle release, suggesting the presence of a fraction of HCV particles or their replication machinery within autophagosomes [70]. However, the impact of the HCV infection on autophagy regulation varies at different stages. In the early stages, the upregulation of Rubicon, a negative regulator of the autophagosome-–ysosome fusion, inhibits autophagic flux, suggesting that HCV replication may rely on autophagosome accumulation [71]. Additionally, impaired autophagy degradation promotes the release of EVs and exosomes. Interestingly, blocking the release of EVs and exosomes and significantly inhibiting viral replication does not compromise the viability of host cells. Furthermore, inhibiting extracellular vesicle release triggers interferon secretion, indicating that the release of EVs serves as an innate immune evasion mechanism that facilitates persistent HCV infection [72]. Consequently, inhibiting the release of EVs represents a potential antiviral strategy for the treatment of HCV and other emerging RNA virus infections.

#### 4.1.3. Other RNA Viruses

Caprine parainfluenza virus type 3 (CPIV3) is an enveloped, encapsulated virus with a single-stranded negative-sense RNA genome [73]. It is a significant respiratory pathogen in goats [74]. Studies have demonstrated that the CPIV3 infection leads to an increase in exosome secretion and the incorporation of viral proteins and RNA into exosomes. These exosomes can transfer CPIV3 genetic material to recipient cells, establishing productive infection and facilitating viral replication. Functional annotation analysis identified the autophagy signaling pathway as the primary pathway involved based on miRNA target predictions. These findings further confirm that CPIV3 exosomes inhibit autophagy, with miR-126–3p_2 identified as a crucial regulator of this process [75]. Inhibition of autophagy may be a key factor contributing to the promotion of effective CPIV3 replication mediated by exosomes.

Flaviviruses, such as Dengue, West Nile, Japanese encephalitis, and Tick-borne encephalitis virus, are significant emerging human pathogens that affect millions of people worldwide [76]. Among them, the dengue virus (DENV) is the most important mosquito-borne flavivirus, endemic primarily in tropical and subtropical regions, and causing an estimated 390 million infections annually, including approximately 96 million symptomatic cases [77]. The DENV infection disrupts endo-lysosomal trafficking and impairs autolysosome formation. Studies have shown that DENV-infected cells exhibit a significant decrease in the colocalization of LC3-positive vesicles with Lamp2-positive vesicles compared to uninfected cells. Furthermore, DENV infection leads to an increase in the number of autophagic vesicles that are not associated with lysosomes. Considering that both autophagy and endo-lysosomal pathways converge at lysosomes for the degradation of intra- and extracellular material, the impact of DENV on endo-lysosomal trafficking was investigated. Using a fluorogenic substrate (DQ-BSA), which measures the trafficking of cargo to the lysosome, researchers observed a decrease in DQ-BSA signal intensity in DENV-infected cells compared to uninfected cells. This finding suggests that DENV infection significantly impairs endo-lysosomal trafficking and supports the notion of a blockage in fusion between autophagosomes/endosomes and lysosomes [78].

Rift Valley Fever virus (RVFV) is an enveloped, segmented, negative-sense RNA virus that replicates in the cytoplasm of host cells [79]. Its genome consists of L, M, and S segments, and it is primarily transmitted by mosquitoes. RVFV can cause severe illness in humans and ruminants and is one of the most extensively studied Bunyaviruses [80]. When naive immune cells (U937 monocytes) and non-immune cells (HSAEC) are treated with exosomes (EXi-RVFV) released from RVFV-infected cells, a potent RIG-I-dependent activation of IFN-B occurs. This robust antiviral response triggers the activation of autophagy in the recipient cells, leading to resistance against subsequent viral infections. This discovery unveils a novel mechanism by which exosomes released from infected cells (EXi) protect the host. EXi activates RIG-I and induces autophagy in recipient cells, including monocytes, through IFN activation. Since monocytes serve as hosts for RVFV replication, the EXi-RVFV-induced activation of autophagy in monocytes may impede or halt the spread of the virus within infected organisms [81]. Hence, the interplay between exosomes and autophagy plays a crucial role in the prevention and control of Rift Valley Fever virus.

### 4.2. DNA Viruses

Apart from enveloped RNA viruses, the interplay between exosomes and autophagy is also implicated in the infection of several enveloped DNA viruses, including HBV, human cytomegalovirus (HCMV), and herpes simplex virus 1 (HSV-1). These enveloped DNA viruses exploit the crosstalk between exosomes and autophagy to enhance viral infection, replication, and release, thereby promoting the spread of infection. 

#### 4.2.1. Hepatitis B Virus (HBV)

Hepatitis B virus (HBV) is an enveloped DNA virus with a partially double-stranded genome, and it is the leading cause of acute and chronic hepatitis B in humans [82]. Hepatitis B remains a prevalent infectious disease worldwide, with chronic hepatitis B (CHB) patients facing a high risk of cirrhosis, liver failure, and hepatocellular carcinoma (HCC), resulting in approximately 1 million deaths annually [83]. 

It has been discovered that HBV particles, as well as subviral particles (SVPs), can be secreted through the ESCRT-dependent MVB pathway [84,85]. Inhibiting the fusion of autophagosomes (APs) with lysosomes can promote the secretion of SVPs and virions [86,87], indicating that APs might fuse with other compartments to release HBV products. However, autophagosomes have the capability to fuse with late endosomes/MVBs, resulting in the formation of amphisomes. These amphisomes then fuse with lysosomes for degradation of their contents or fuse with the plasma membrane to release their luminal vesicles (exosomal precursors) and cargo into the extracellular space [39,88]. In certain studies, the induction of autophagy using the endoplasmic reticulum stress inducer Tunicamycin (TM) in liver cancer cells revealed that HBV promotes its replication and secretion through the crosstalk between AP-MVB. This AP-MVB crosstalk serves as an essential secretion pathway for HBsAg/SVPs and capsids [89].

#### 4.2.2. Herpes Simplex Virus-1 (HSV-1)

Herpes simplex virus-1 (HSV-1) is a double-stranded linear DNA virus enclosed within a capsid [90]. It is responsible for various clinical manifestations, including mucosal skin lesions, keratoconjunctivitis, encephalitis, and respiratory infections, and is highly prevalent in humans [91]. During infection, viral DNA replication, gene transcription, and nucleocapsid assembly occur within the nucleus. Once assembly is complete, progeny virions initially bud from the nuclear envelope into the cytoplasm, acquiring a lipid envelope. Subsequently, they undergo a secondary budding process at the plasma membrane, obtaining a second envelope before being released into the extracellular environment [92]. Studies have identified the presence of virions within vesicles released by infected cells. Although the precise mechanism by which HSV-1 targets these vesicles is not fully understood, the presence of the autophagy marker LC3-II in vesicles isolated from HSV-1-infected cells suggests the potential involvement of the autophagy pathway in vesicle-mediated HSV-1 transmission [93]. These findings indicate that HSV-1 may exploit vesicles for virus transmission, while autophagy influences the export and spread of HSV-1 through interactions with EVs. Furthermore, the HSV-1 protein ICP34.5 targets BECN1 to inhibit the initiation of autophagosome formation or the fusion of autophagosomes with lysosomes, thereby promoting viral replication [94]. Conversely, BECN1 inhibits the excessive production of interferon (IFN) and facilitates the autophagic degradation of intracellular pathogens such as HSV-1 [95]. The interaction between viral proteins and BECN1 appears to contribute to increased viral replication [96].

#### 4.2.3. Other Enveloped DNA Viruses

The morphology of human cytomegalovirus (HCMV) closely resembles that of HSV [97]. HCMV infects a significant portion of the global population and is a major cause of congenital disabilities [98]. UL32-GFP fibroblasts containing MVBs were found to originate from the conventional endocytic pathway and the classical exosome release pathway. However, UL32-GFP-containing MVBs in primary human microvascular endothelial cells (HMVECs) originate from the early biosynthetic pathway and utilize the early Golgi-LAMP1-associated atypical secretory autophagy pathway. These findings highlight cell type-specific differences in membrane transport within the host pathway utilized by HCMV, potentially reflecting distinct viral release mechanisms [99]. Further investigation is required to understand the specific mechanism by which HCMV proliferates through the secretory autophagy pathway.

Varicella zoster virus (VZV) is a human herpesvirus that causes primary infection with varicella, leading to herpes zoster [100,101]. Clinical manifestations of VZV infection include rash, blisters, fever, pain, sore throat, and headache [102]. Studies have demonstrated upregulation of autophagy in fibroblasts and human melanoma cells 48 h after VZV infection, although no specific protein has been identified as a precise activator of the autophagy pathway [103]. Upregulation of autophagy after VZV infection has been observed in fibroblasts, particularly MRC-5 cells, as well as in primary cells, MeWo cells, and keratinocytes [103,104,105]. Additionally, VZV glycoprotein transfection in HeLa cells led to increased endoplasmic reticulum and LC3 formation [106]. Research conducted on human skin confirmed a significant increase in autophagic vesicles during VZV infection, [104], suggesting that autophagy plays a crucial role in the pathogenesis of the disease [107]. Cells deficient in autophagy exhibited a significant reduction in viral titers, indicating that autophagy contributes to promoting VZV exocytosis. Colocalization of viral particles with RAS-related protein (Rab)11 was also observed [108]. Other studies have shown that in healthy cells, viral capsids cluster in large vacuoles containing numerous virus particles. Conversely, autophagy-deficient cells display small vacuoles containing only a single virus particle [104]. This observation might explain the potential escape mechanism employed by VZV, as it exists within autophagosomes and fuses with late endosomes, enabling the virus to evade degradation and be transported to the plasma membrane for cell exit. Overall, most studies support the notion that autophagy plays a beneficial role in promoting cell survival and facilitating the VZV life cycle. A comprehensive understanding of the intricate interplay between VZV, autophagy, and exosome mechanisms necessitates the integration of various approaches.

Furthermore, autophagy has also been utilized by pseudorabies virus (PRV) and duck enteritis virus (DEV). Infections caused by PRV and DEV not only induce an increase in autophagy flux but also demonstrate that enhanced autophagy promotes viral replication, while inhibition of autophagy impedes replication [109,110,111].

## 5. Conclusions and Prospect

Exosome biogenesis and autophagy are vital for maintaining cellular homeostasis and reducing cellular stress. Current evidence suggests that these processes involve intricate crosstalk between autophagy and exosomes. Exosomes possess the ability to modulate intracellular autophagy through diverse signaling pathways and play a crucial role in various diseases. Similarly, autophagy governs the formation and release of exosomes both inside and outside cells. It plays a significant role in physiological and pathological processes, including enveloped virus infections, wherein it exerts a substantial influence on the virus. This influence encompasses viral nucleic acids, proteins, and even viral particles, which can be secreted into the body or exploit the autophagy pathway to evade the host cell’s immune system, thereby facilitating virus propagation and dissemination.

Despite its significance, the role of this crosstalk in enveloped virus infections has been inadequately investigated, highlighting the need for further research. Therefore, gaining a comprehensive understanding of the molecular mechanisms underlying the interaction and the specific processes involved in exosome biogenesis and secretory autophagy during enveloped virus infections can provide a novel perspective for inhibiting virus replication, preventing virus transmission, developing viral vaccines, and designing targeted antiviral drugs. With deeper exploration into the mechanisms of enveloped virus infections, we anticipate the development of strategies that can effectively prevent and inhibit such infections by targeting the crosstalk between exosomes and autophagy.

Both exosome biogenesis and autophagy are critical for maintaining intracellular equilibrium and mitigating cell stress. Accumulating evidence suggests that these cellular responses rely on interactions between autophagy and exosomes, which are indispensable for the progression of various diseases, including viral infections. Viruses may exploit autophagosome crosstalk to enhance their replication and release. Investigating novel mechanisms for the release of extracellular cargo from infected cells holds promise for host protection. Furthermore, exosomes and their microRNAs are anticipated to serve as minimally invasive biomarkers and targets for antiviral research and the development of novel vaccines against viral infections.

Exosomes regulate intracellular autophagy through multiple signaling pathways, while autophagy modulates the biogenesis and release of both intracellular and extracellular exosomes, which holds significant physiological and pathological implications. By acquiring a better understanding and conducting in-depth studies on the molecular and regulatory mechanisms governing their interaction in disease progression, we can enhance our ability to prevent and control diseases in the near future. 

## Figures and Tables

**Table 1:** Differences between exosome and autophagy.

	Exosomes	Autophagy
Inducement	Cytokine	Nutrient deficiency, growth factor deficiency, ROS, etc.
Initiation Contents	Endocytosis Proteins, lipids, and nucleic acids	ULK1, mTORC1, PI3K Protein aggregates, lipids, carbohydrate or organelles
Sorting/extension	ESCRT	VPS34, ATGs, Beclin-1,PE, LC3B
Intermediate organelle Regulatory mechanism Shared intermediate organelles	MVBs, ILVs ISGylation Amphisome	Autophagosomes mTOR Signaling Pathway Amphisome
Fusion and Degradation	Lysosome	Lysosome
Products	Exosomes	Sugars, lipids, and amino acids
Function	Removing harmful substances from cells or transmitting cell information	Maintaining cell survival

## Data Availability

All data generated or analyzed during this study are included in this published article.

## Abbreviations

MVBs	multivesicular bodies
ILVs	intraluminal vesicles
ESCRT	endosomal sorting complex required for transport
SIMPLE	small integral membrane protein of lysosome/late endosome, also known as lipopolysaccharide-induced TNF-α factor (LITAF)
Ago	argonaute, a major member of the RNA-induced silencing complex
ATGs	autophagy-related proteins
PE	phosphatidylethanolamine
mTOR	mammalian target of rapamycin
MAP-LC3	microtubule-associated protein light chain 3
GABA-RAP	γ-aminobutyric-acid-type-A-receptor-associated protein
GATE-16	Golgi-associated ATPase enhancer of 16 kDa
BECN1	Beclin1
VPS4A	Vacuolar Protein Sorting 4A
EVs	extracellular vesicles
Lamp2	Lysosomal Associated Membrane Protein 2
EXi	exosomes released from infected cells
APs	autophagosomes
SVPs	subviral particles
HBsAg	hepatitis B surface antigen
